# An Early Warning Mobile Health Screening and Risk Scoring App for Preventing In-Hospital Transmission of COVID-19 by Health Care Workers: Development and Feasibility Study

**DOI:** 10.2196/27521

**Published:** 2021-12-17

**Authors:** Ronald Mbiine, Cephas Nakanwagi, Herve Monka Lekuya, Joan Aine, Kawesi Hakim, Lilian Nabunya, Henry Tomusange

**Affiliations:** 1 Makerere University College of Health Sciences Kampala Uganda; 2 Mulago National Referral Hospital Kampala Uganda

**Keywords:** mHealth, risk score for Covid-19, Africa, mobile health, digital health, pandemic, COVID-19, COVID, screening tool, healthcare workers, transmission, warning system

## Abstract

**Background:**

Hospitals have been identified as very high-risk places for COVID-19 transmission between health care workers and patients who do not have COVID-19. Health care workers are the most at-risk population to contract and transmit the infection, especially to already vulnerable patients who do not have COVID-19. In low-income countries, routine testing is not feasible due to the high cost of testing; therefore, presenting the risk of uncontrolled transmission within non–COVID-19 treatment wards. This challenge necessitated the development of an affordable intermediary screening tool that would enable early identification of potentially infected health care workers and for early real time DNA–polymerase chain reaction testing prioritization. This would limit the contact time of potentially infected health care workers with the patients but also enable efficient use of the limited testing kits.

**Objective:**

The aims of this study are to describe an early warning in-hospital mobile risk analysis app for screening COVID-19 and to determine the feasibility and user-friendliness of the app among health care workers.

**Methods:**

The primary result of this research project was the development of a mobile-based daily early warning system for in-hospital transmission of COVID-19. Overall, the Early Warning System for In-Hospital Transmission of COVID-19 (EWAS) mobile app was found to be feasible, with over 69% of the health care workers having logged more than 67% of the required times. Over 93% of the participants reported that the tool was easy to use.

**Results:**

The primary result of this research project was the development of a mobile-based daily early warning system for in-hospital transmission of COVID-19. Overall, the Early Warning System for In-Hospital Transmission of COVID-19 (EWAS) mobile app was found to be feasible, with 69% of the health care workers (69/100) having logged more than 67% of the required times. Of the 100 participants, 93 reported that the tool was easy to use.

**Conclusions:**

The EWAS mobile app is a feasible and user-friendly daily risk scoring tool for preventing in-hospital transmission of COVID-19. Although it was not designed to be a diagnostic tool but rather a screening tool, there is a need to evaluate its sensitivity in predicting persons likely to have contracted COVID-19.

## Introduction

COVID-19 continues to present a serious national and global challenge [1]. In Uganda, the disease is now at the community transmission stage, implying that all mitigation measures such as contact tracing are no longer feasible.

One of the key identified areas of superspread is the hospitals where patients who do not have COVID-19 go to seek care [2,3]. Health care workers, by the nature of their work, are at the highest risk of contracting and transmitting COVID-19 [4]. Its estimated that over 10,000 health care workers in Africa have been infected with COVID-19 [5]; meanwhile, a significant number of vulnerable patients contract COVID-19 while admitted to hospitals for other ailments [6]. To minimize the in-hospital transmission of COVID-19, especially by health care providers who interact with several patients, it would be necessary to conduct routine DNA–polymerase chain reaction (PCR) tests for COVID-19 among these health care providers. The cost of a DNA-PCR COVID-19 test, which is approximately US $50 in Uganda [7], coupled with the 24- to 48-hour turnaround time for the results makes this test an unsustainable screening method for early prevention of in-hospital disease transmission in the medium term, especially in low-income country hospital settings.

This therefore presents a great challenge in early detection of COVID-19, creating the need for an intermediary screening tool that can easily identify potentially sick health care workers based on a daily log of their symptoms and contact history.

The Early Warning System for In-Hospital Transmission of COVID-19 (EWAS) is a mobile health (mHealth)–based app that is designed purposefully to address this gap. This application enables the daily screening and early identification of potentially infected health care workers for prioritization in testing; this will enable better use of the limited DNA-PCR test kits, as priority will be given to testing those with a high risk score. This reduces the need to conduct regular mass DNA-PCR testing of all staff, which is neither feasible nor sustainable. The mobile app thus creates an intermediary risk-scoring tool that is self-administered and very simple, and it could quickly identify a potentially sick health care worker long before they suspect that they have COVID-19 or obtain their test result.

Owing to the ambiguous nature of COVID-19 symptoms, infected persons are usually initially skeptical about having contracted COVID-19; it is only after they have developed the classic symptoms that they may proceed to self-isolate and undergo a test.

Often during this vague prodromal period, the virus is already transmissible, and the unsuspecting health care worker may still be delivering care. With a daily screening mobile app, the presence of these vague symptoms may be identified, enabling the instillation of the necessary mitigation measures on the ward.

The mobile app also has the ability to predict cluster epidemics long before they would be observed, as a collective high score among multiple health care workers on one of the wards would imply this possibility.

This tool therefore complements DNA-PCR testing as an adjunctive intermediary screening tool and does not replace or intend to replace the existing testing as recommended by the Ministry of Health of Uganda. Therefore, the intention of the study is not to see how sensitive the app is in diagnosing COVID-19; as this was never the purpose of its development, there is no intention to evaluate its sensitivity or specificity.

The primary research question for this study was to determine the feasibility and acceptance of using the EWAS mobile app in reducing hospital transmission of COVID-19.

We hypothesize that the EWAS app is a feasible and acceptable intermediary screening tool for preventing transmission of COVID-19 on non–COVID-19 treatment wards.

The objectives of this study where therefore to create an intermediary daily early warning COVID-19 risk scoring screening mobile tool and evaluate its feasibility and acceptability of use on the clinical wards in Mulago Hospital.

The intended aim would be to prioritize health care workers with high risk scores for DNA-PCR testing and timely intervention. In this way, the exposure time of potentially infected health care workers to patients would be significantly reduced, thus minimizing the in-hospital transmission of COVID-19 on general and non–COVID-19 treatment units.

In this study, first, we describe the mobile app in detail, and we subsequently describe the feasibility and acceptability findings for its use.

## Methods

This study constituted of two phases. The first phase was the design and development of the software app, and the second phase included the prospective piloting of the mobile app.

### Study Setting

The study was conducted in the Directorate of Surgical Services of the Mulago National Referral Hospital. This is the largest national referral hospital in Uganda, based in Kampala.

The Directorate of Surgery comprises five surgical departments: neurosurgery, gastrointestinal surgery, cardiothoracic surgery, accident and emergency, and breast and endocrine.

These wards treat surgical conditions and are not designated COVID-19 treatment units; the average monthly patient turnover is 300.

### Study Population

The study participants included health care workers who directly participated in patient care in the different departments in the Directorate of Surgical Services. These include surgeons, surgical residents, nurses, and intern physicians.

### Sampling and Sample Size Estimation

This aim of this study was to assess the feasibility of using this app, and it is the first of its kind.

Because it was a pilot study, the sample size was based on the convenience of a workable number for the stipulated study period of 1 month. Therefore, 20 participants were randomly selected from each of the five departments in the Directorate of Surgery to constitute a sample size of 100 participants.

To be eligible for inclusion in the study, participants were required to have a smart mobile phone with internet connectivity. Because the first version of the app was built only for Android phones, participants with iPhones were excluded from the study.

### Outcome Variables

The primary outcome variables for the study were as follows:

1. Feasibility of regular use: For this study, feasibility of regular use was determined as the mean number of days on which users logged their risk scores into the mobile app. Logging at least 20 out of the 30 daily scores was ranked as “feasible for regular use.”
2. User-friendliness: This was defined based on the participants’ rating of how easy it was to understand and use the mobile app. This was based on a 5-point Linkert scale, with the highest score of 5 for “very user-friendly” and zero for “not user-friendly.”

### Study Procedure

Ethical approval and administrative clearance were obtained from the Mulago National Referral Hospital ethics review board. Upon clearance, the project was implemented in 2 phases. The first phase was the app development, and the second phase was the feasibility assessment.

### Application Development

The development of the EWAS mobile app comprised two subphases: (1) risk assessment tool and algorithm development and (2) software design and development.

### Risk Screening Tool and Algorithm Development

To develop a standard risk assessment tool, we identified the World Health Organization (WHO) [8] and Ministry of Health of Uganda [9] guidelines for the clinical case definition of COVID-19.

**Figure 1:**
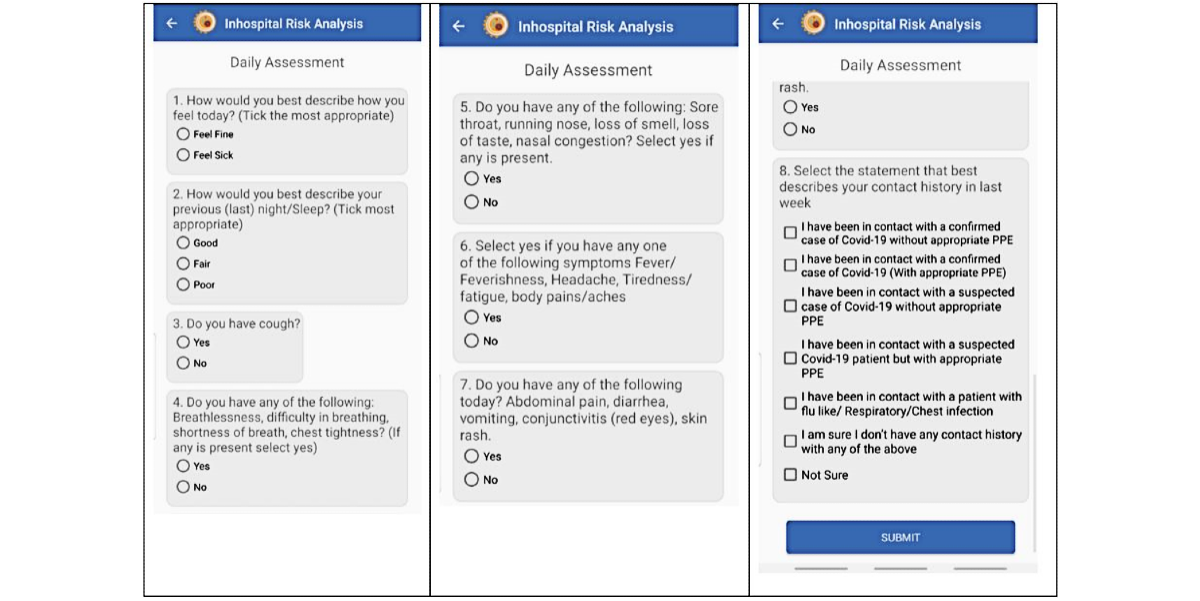
Screenshots of the risk assessment tool in the Early Warning System for In-Hospital Transmission of COVID-19 (EWAS) app.

Based on the above case definitions, we developed an 8-question risk assessment and scoring tool comprising multiple-choice answers, of which the participant could only select a single answer for each question (see Figure 1). This was done to minimize user fatigue from the long examination-style assessment tools that are commonly used for screening.

Each selected answer was assigned a score based on whether it comprised a positive symptom for COVID-19.

The 8 questions were grouped into 2 sections; section I comprised the symptom score, which was based on 7 questions evaluating the user’s general health condition as well as the presence of COVID-19–like symptoms. The overall maximum score for section I was 155, with the minimum score being 30.

Section II comprised the contact score, consisting of 1 multiple-choice question describing possible scenarios for the contact history of the user in the last week. Each answer was weighted, with the maximum score being 50 and the minimum being 15.

To include potential contact with asymptomatic carriers, even when the participant was sure that they had not been in contact with a suspected COVID-19–positive patient, they could not be awarded a score of 0.

To avoid falsely alarming high risk scores, each of the 8 questions was weighted based on how prevalent the given symptoms were among patients with COVID-19 in Uganda [10].

Section I and section II each contributed 50% to the overall risk score, which was calculated as the percentage of the participant score relative to the total score.

Hence, to calculate the overall patient risk score, the patient symptom score and the contact score would be entered into the following formula:

Patient overall risk score = [(symptom score ÷ 155) × 50] + [(contact score ÷ 50) × 50]

**Figure 2:**
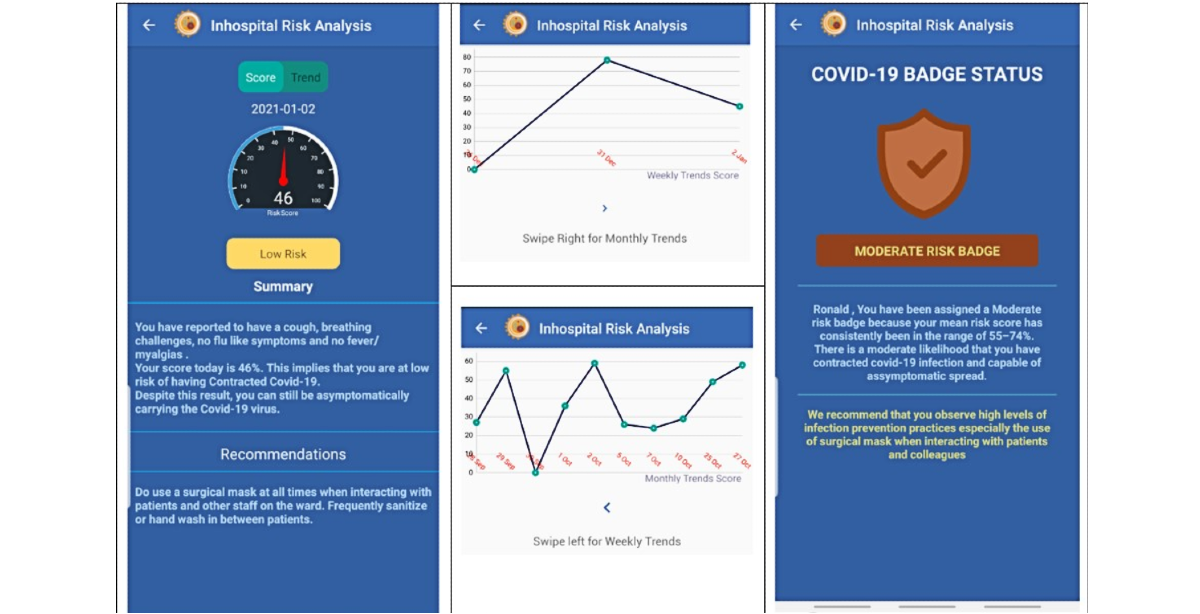
Screenshots showing the daily risk score and weekly risk badge in the Early Warning System for In-Hospital Transmission of COVID-19 (EWAS) app.

Based on the daily input, a daily risk score would be awarded to the participant (Figure 2, left).

The second step was to calculate a trend analysis of the daily logged risk score.

Based on the weekly trends of the risk scores displayed in a graphical pattern (Figure 2, center), the participant was issued a weekly risk badge (Figure 2, right). The risk badge would generally reflect the risk status of the patient regarding their COVID-19 symptoms and contact score. The intention of the risk badge was to identify participants who had a high-risk badge and prioritize them for a weekly DNA-PCR test.

Hence, if a participant consistently had a high risk score through the week, their risk badge would identify them as high risk and indicate the necessity for urgent DNA-PCR testing.

The app also identified clusters of participants with high-risk badges, and if these were on one ward, this would quickly highlight the affected ward as having potential for a cluster outbreak within the hospital; this feature was implemented with the intention of enabling timely intervention before the lives of patients and other health care workers were placed at high risk.

Once the above risk assessment tools and algorithms were completed, they were reviewed by a clinic epidemiologist and a physician who was directly participating in the treatment of patients with COVID-19 at Mulago National Referral Hospital, and after they cleared the review process, ethical clearance was obtained from the Research and Ethics Review Board of Mulago Hospital (protocol number MHREC 1901).

Subsequently, the software developer team undertook the development of the mobile app with multiple pretests to ensure good end-user experience and relevancy.

To improve user-friendliness, the app’s risk assessment tool was designed to be as simple as possible, with minimal dropdown menus and no direct user input.

Because English is the official language of communication between health care workers while delivering care, the app was developed to be used only in English, and all the health care workers were sufficiently proficient in speaking, reading, and writing in the English language.

The app was piloted for 1 week among 10 participants, and the user feedback was used to make final adjustments prior to the official launch [11].

The app was later published on the Google Play store as “In-hospital Early Risk Analysis for COVID-19” [12].

### Availability and Requirements

The availability and requirements of the app are listed in Textbox 1.

**The availability and requirements of the Early Warning System for In-Hospital Transmission of COVID-19 (EWAS) app.**

**Project name**: Early Warning System for In-Hospital Transmission of COVID-19 (EWAS)

**Project home page**: https://ewasinhospital.org/ [11]

**Google Play address**: https://play.google.com/store/apps/details?id=org.ewasinhospital.app.mobile [12]

**Operating system**: Android, platform independent

**Programming languages**: Java, Python, Django

**Other requirements**: Android 5+, Python 3, MySQL

**License**: none

**Any restrictions to use by nonacademics**: none

### Study Implementation

Following the selection of the 100 participants, user training was provided and the mobile app was installed on their devices. The participants were required to register in the software app prior to use, and their profession (position) and ward were captured. The participants were instructed to use the software on a daily basis for a period of 30 days. Participants were to log their daily risk scores and weekly risk badges and to share them with the research assistants. In the event that they had a high-risk badge, they were advised to contact COVID-19 surveillance for further assessment.

At the end of the study duration, the participants were required to answer a questionnaire to evaluate their user experience. The participants were also invited to provide information on how the app could be made more relevant in subsequent versions.

## Results

### Participant Demographics

In total, 100 participants were enrolled in the study, comprising 26 (26%) junior house officers (intern physicians), 28 (28%) nurses, 24 (24%) senior house officers (surgical residents), and 22 (22%) surgeons.

### Distribution of Staff in the Different Surgical Unit

The participants were distributed in different units, as shown in Table 1, with the majority being in the gastrointestinal surgical unit.

**Table 1:** Ward distribution of the health care workers who participated in the study (N=100).

Unit	Participants, n (%)
Accident and emergency	20 (20)
Breast and endocrine	18 (18)
Cardiothoracic surgery	16 (16)
Gastrointestinal surgery	26 (26)
Neurosurgery	20 (20)

### Participant Mean Risk Scores Per Week

In the first week, 24% of the participants (24/100) had a high risk score, while in the second, third, and fourth weeks, 26, 19, and 23 of the 100 participants reported a high risk score, respectively (Table 2).

Table 3 shows the proportions of participants with high risk scores, as in, participants with risk scores greater than 75% in each week.

**Table 2:** Weekly mean risk scores of the participants.

Week	Risk score, mean (SD)
1	51.5 (14)
2	53 (14.68)
3	54.27 (14.06)
4	50.32 (13.92)

**Table 3:** Participants with high weekly risk scores per week (N=100).

Week	High risk scores, n (%)
1	11 (11)
2	12 (12)
3	14 (14)
4	10 (10)

### Feasibility of Regular Use/Compliance

The mean number of days that were logged over the 30-day period was 21.97 (SD 5.99); 69% of the participants (69/100) logged their data more than 20 times, which demonstrates high compliance and feasibility of use of the mobile app.

### Comparison of Regular Use Between Nurses, Junior House Officers, Senior House Officers, and Surgeons

Overall, the intern physicians demonstrated more use and compliance, with 73% (19/26) having logged and registered their scores on at least 20 of the 30 days; this was followed by the nurses, with 71% compliance (20/28). The surgeons had the lowest compliance among all the participants (Table 4).

**Table 4:** Feasibility of regular app use by profession.

Professional background	Compliance, n (%)	
	Low	High
Junior house officers (n=26)	7 (27)	19 (73)
Senior house officers (n=24)	8 (33)	16 (67)
Nurses (n=28)	8 (29)	20 (71)
Surgeons (n=22)	8 (36)	14 (64)
Total (N=100)	31 (31)	69 (69)

### User Experience

Of the 100 participants, 93 described the app as very easy to use, while 3 described the app as easy to use and 4 described the application as not easy, difficult, or very difficult to use. User errors or app crashing was reported by only 2 of the 100 participants, with 98 reporting no history of app crashes or errors.

The COVID-19 DNA-PCR testing rates were very low in the month under review, with only 7% (7/100) of the staff having undertaken a COVID-19 test.

Of the 100 participants, 74 (74%) were willing to continue using the app even after the end of the study.

### Efficacy in Predicting Positive COVID-19 Infection

At the time of the study implementation, the project had not been incorporated into the Ministry of Health protocols for managing COVID-19; therefore, these protocols could not independently inform the need to request a COVID-19 PCR test. Second, the unavailability of the DNA-PCR test for regular disease screening, coupled with the associated cost of $50 USD for each test, resulted in very low testing rates among the health care providers, with less than 10 having undertaken the DNA-PCR test during the period under review. The study could not therefore compare the efficacy of the tool in predicting potentially high-risk participants.

The primary intended outcome of this research project was to create an intermediary early warning tool that would help identify symptomatic health care workers to prevent in-hospital transmission of COVID-19 while also identifying health care workers who would require a DNA-PCR test without necessarily having to routinely perform mass testing of all health care workers, which is costly and unsustainable.

With the mobile app in place, health care workers are now able to obtain a daily risk score that is objective and quantifiable. This daily symptom score, when used, can help identify the disease process long before the health care worker would think that they had COVID-19, hence limiting the contact time of a potentially infected health care worker with a patient.

The health care worker can also access a graphical representation of their risk trends for the past week and month. This risk trend is the basis of the risk badge (Figure 2) that is assigned to the health care worker every after 5 days. The risk badge therefore forms the basis for the need for further analysis and intervention including DNA-PCR testing and mitigation measures, such as self-isolation.

The software also enables notification and messaging, through which an affected health care worker can send out an urgent request for support from the COVID-19 treatment team, including a request for a dispatch team. The app also comprises a repository for maintained and tracking COVID-19 DNA-PCR test results, which the user uploads onto their database.

## Discussion

### Principal Findings

In-hospital transmission of COVID-19 among health care workers and patients is still a recognized challenge in prevention of COVID-19 transmission [13], especially to already vulnerable populations. The most ideal way of minimizing transmission would be routinely performing point-of-care DNA-PCR testing of all health care workers and patients on various non–COVID-19 treatment units, such as surgical wards [14]. In the study period, the user participants had a <7% (7/100) testing rate for COVID-19. This further illustrates the unsustainability of DNA-PCR testing as a regular screening tool for preventing COVID-19 infection [15,16]. In our study setting, the prohibitive US $50 out-of-pocket cost reduced the likelihood of regular testing as an alternative for preventing in-hospital transmission of COVID-19 [7].

Similarly, regular routine DNA-PCR testing is globally recognized as unfeasible, especially in low- and middle-income countries; therefore, in-hospital transmission of COVID-19 remains a substantial challenge [17,18].

Therefore, there was a need for an easily accessible self-administered daily risk scoring tool, which led to the development of this mHealth solution. One of the important concerns related to mHealth apps is whether they are feasible and can consistently be used unsupervised on a daily basis.

Overall, we found that the EWAS mobile app was feasible to use, based on the fact that the consistent use by 69% of the participants was similar to findings in other settings where digital screening tools were adapted for early detection of COVID-19 [19,20].

Interestingly, we found that intern physicians and nurses were more likely to consistently use the app when compared to surgical residents and surgeons. Unsurprisingly, digital utilization is more commonly adapted among younger medical professionals [21]; this explains the comparably high use among junior house officers, including junior physicians. Similarly, nurses have been described worldwide as embracing and using digital health applications [22,23]; this finding is consistent with the increased uptake and use of the mobile app by nurses in our study population. Intern physicians and nurses spend more contact time on the wards and are hence more likely to contract COVID-19 infection. Their increased ward contact in comparison to that of surgeons could also explain why the surgeons’ consistency of use was the lowest.

Our study demonstrated that a mobile app for daily risk monitoring of COVID-19 is feasible and could consistently be used use a daily risk scoring tool for preventing in-hospital transmission of COVID-19.

The EWAS app for in-hospital transmission of COVID-19 can therefore be implemented as a daily screening tool to reduce the transmission rates on hospital wards by identifying health care workers who are potentially infected with the disease and therefore may be transmitting it. The tool is not designed to replace or be a diagnostic tool but to screen health care workers who may be prioritized for testing.

At the time of developing and implementing this project, there was no established local screening tool that would assign a risk score to a participant based on their symptoms.

Patients would be evaluated for COVID-19–defining symptoms, and if these were classic based on the WHO [8] and Uganda government [9] guidelines, a test would then be recommended. These evaluations were not intended to identify potentially infected persons but identify patients for treatment.

Therefore, there was no comparative gold standard risk assessment screening tool that our app could be compared to; hence, it was necessary to perform a pilot study.

In highly transmissible diseases such as COVID-19, mHealth apps have been described to significantly improve disease screening and symptom monitoring [24-26]. Daily screening tools have an important role to play in preventing COVID-19 [27,28], as they may identify an infected person before they actually become suspicious of being infected.

In China, mHealth apps were shown to significantly reduce disease transmission [29]; however, these apps were designed for use in community settings. In Sweden, a similar mHealth app that tracks logistic use, including personal protective equipment, and patient care in 5 hospitals has been developed and is in use [30]. Overall, use of mHealth apps in disease tracking and symptom monitoring as an adjunct to existing guidelines in the management of COVID-19 is increasing [31].

In Africa, there has equally been a growing interest in using digital health technologies as surveillance or treatment monitoring tools [32]; however, no studies evaluating the use of mHealth solutions in preventing in-hospital transmission have been described.

The availability of this mHealth self-administered screening and risk assessment software in low-income countries may be relevant in the prevention of day-to-day disease transmission. This tool represents a first-line in-hospital active screening tool that is capable of identifying potentially infected health care workers before they transmit the illness. Without this app in our health care system, in-hospital transmission is essentially left to the discretion of the affected health care worker, and this represents a potential loophole for limiting disease transmission.

It should be noted that the EWAS mobile app does not replace testing or any other established guidelines in the diagnosis of COVID-19; however, it can be used in combination as an intermediary adjunct with these existing guidelines to increase the likelihood of minimizing the in-hospital transmission of COVID-19.

By being able to identify the health care workers most likely to be infected, the EWAS app can therefore maximize the potential benefits in screening and prioritizing the limited testing resources of DNA-PCR for health care workers in large hospital settings, such as the Mulago National Referral Hospital.

Despite the advantages of the EWAS app, health care provider compliance remains a great challenge, especially as the pandemic progresses. As the general fear associated with COVID-19 has waned, so has the compliance with the majority of established standard operating procedures, including compliance with the EWAS app.

This complacency is partly responsible for the recent spikes in the spread of COVID-19; however the increasing spread calls for more aggressive campaigns and adaption of all mitigating measures, including the adoption of the EWAS tool in non–COVID-19 treatment units.

Moreover, for all health care workers to successfully use these tools, they would need to have access to a smartphone as well as a mobile data connection. In Uganda, smartphone coverage is approximately 42% [33], with over 70% smartphone coverage in urban centers. In Mulago Hospital, based on a preliminary survey, smartphone coverage was approximately 90% among health care workers; however, despite this good coverage, the ability to fully use the smartphones, including installation of the app and registration of the user, required the establishment of support services. To a great extent, this may limit the realization of the full potential of integrating mobile apps, including the EWAS app, in disease surveillance and transmission prevention.

### Conclusion

The EWAS mobile app is feasible and can consistently be used as a daily risk-scoring tool to prevent in-hospital transmission of COVID-19 among health care workers.

### Limitations

One of the key limitations of this study is the inability to compare the efficacy of this tool in predicting COVID-19 positivity among the participants. This was due to the inaccessibility to routine testing among participants due to the prohibitive fee of US $50 associated with the DNA-PCR testing.

### Recommendations

We recommend that the tool be further evaluated on a larger scale and in different hospital settings to compare its acceptance and consistence of use. We also recommend that the sensitivity of this tool in predicting a positive infection of COVID-19 compared to DNA-PCR testing be determined. This will help evaluate how good a screening tool the app is and advise on revisions that can be made to greatly increase its sensitivity.

### Data Availability

Data sharing is not applicable to this article, as no data sets were generated or analyzed during the case report.

## Abbreviations

EWAS: Early Warning System for In-Hospital Transfer of COVID-19
MakRIF: Makerere University Research Innovation Fund
mHealth: mobile health
PCR: polymerase chain reaction
WHO: World Health Organization
